# Effect of 3D microstructure of dermal papillae on SED concentration at a mechanoreceptor location

**DOI:** 10.1371/journal.pone.0189293

**Published:** 2017-12-08

**Authors:** Trung Quang Pham, Takayuki Hoshi, Yoshihiro Tanaka, Akihito Sano

**Affiliations:** 1 Department of Engineering Physics, Electronics and Mechanics, Graduate School of Engineering, Nagoya Institute of Technology, Nagoya, Japan; 2 Research Center for Advanced Science and Technology, The University of Tokyo, Tokyo, Japan; 3 Department of Electrical and Mechanical Engineering, Graduate School of Engineering, Nagoya Institute of Technology, Nagoya, Japan; University of Chicago, UNITED STATES

## Abstract

The feeling of touch is an essential human sensation. Four types of mechanoreceptors (i.e., FA-I, SA-I, FA-II, and SA-II) in human skin signalize physical properties, such as shape, size, and texture, of an object that is touched and transmit the signal to the brain. Previous studies attempted to investigate the mechanical properties of skin microstructure and their effect on mechanoreceptors by using finite element modeling. However, very few studies have focused on the three-dimensional microstructure of dermal papillae, and this is related to that of FA-I receptors. A gap exists between conventional 2D models of dermal papillae and the natural configuration, which corresponds to a complex and uneven structure with depth. In this study, the three-dimensional microstructure of dermal papillae is modeled, and the differences between two-dimensional and three-dimensional aspects of dermal papillae on the strain energy density at receptor positions are examined. The three-dimensional microstructure has a focalizing effect and a localizing effect. Results also reveal the potential usefulness of these effects for tactile sensor design, and this may improve edge discrimination.

## Introduction

**Fig 1:**
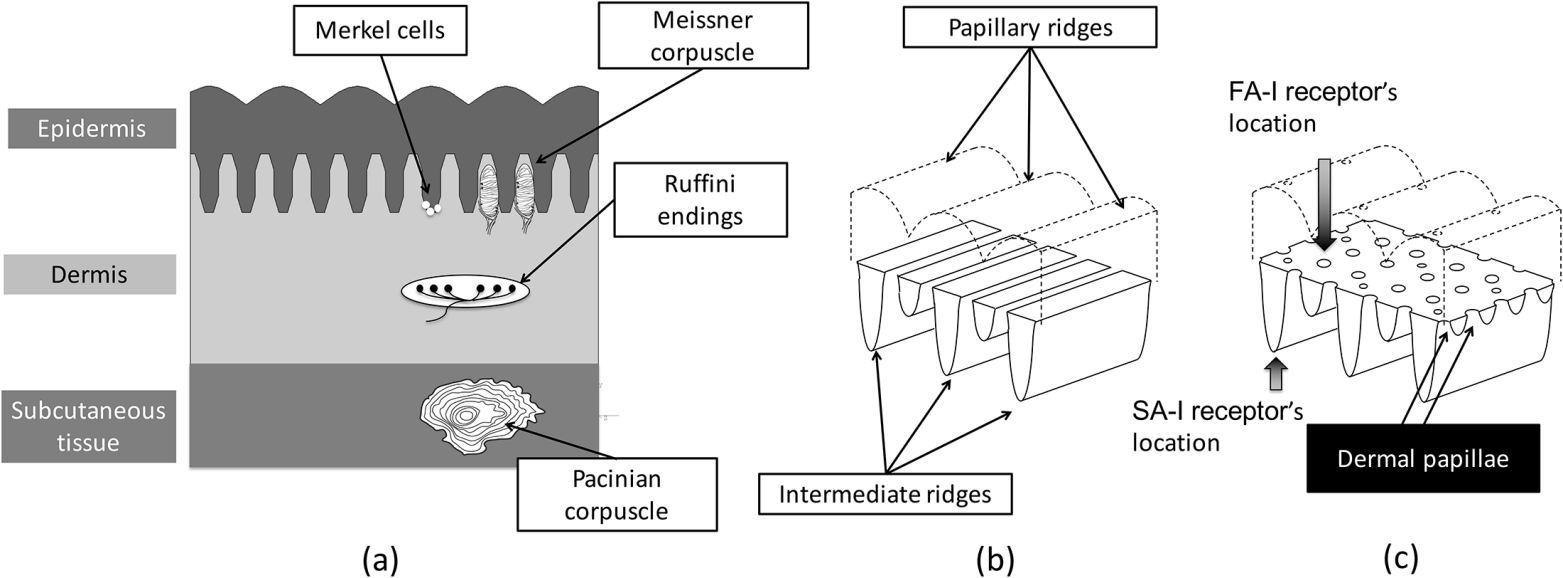
(a) Illustration of the skin cross-section and its mechanoreceptors. (b) Depth assumption of a 2D microstructure and (c) Natural configuration of a 3D microstructure according to [3].

There are four types of mechanoreceptors, namely Pacinian corpuscles, Ruffini endings, Merkel cells, and Meissner corpuscles, which work as haptic sensors for humans. Pacinian corpuscles, Ruffini endings, Merkel cells, and Meissner corpuscles are the end organs of rapidly adapting type-II mechanoreceptors (FA-II) and slowly adapting type-II mechanoreceptors (SA-II) that correspond to SA-I mechanoreceptors and FA-I mechanoreceptors, respectively. They are positioned at different depths of the skin (Fig 1a). Specifically, SA-I and FA-I receptors share the intermediate region between the epidermis and dermis. As shown in Fig 1, the microstructure of the intermediate region between the epidermis and dermis is separated into the following two terms: intermediate ridges that correspond to a typical aspect perpendicular to fingerprints, and dermal papillae that correspond to an aspect parallel to fingerprints. The anatomical components of skin and geometry of ridges are described in [1] and [2].

There is a paucity of research on the mechanotransduction mechanism by which a tactile stimulus is transformed into a neural signal. Finite-element modeling (FEM) is a reliable method for investigating the mechanical properties of skin components and the manner in which they affect mechanoreceptors. Various modeling techniques (from macrostructures to microstructures) were proposed in the biomechanics literature.

In term of macrostructures, a significant model corresponds to the three-dimensional (3D) model of a whole fingertip that was developed based on real human and monkey fingertip geometry by Dandekar et al. [4]. The strain energy density (SED) calculated at the SA-I receptor location of this model was matched with neurophysiological data reported by Phillips and Johnson [5]. A model of the same scale was developed by Gerling et al. [6] with extensive transduction and neural sub-models that convert the SED into neural spikes. The model matched surface deflection in human experiments conducted by Srinivasan [7] and exhibited a good correlation with single afferent responses obtained by Johnson [5] as well as psychophysical results derived by Goodwin [8]. However, fingerprints and other microstructures of skin, such as intermediate ridges, dermal papillae, and fibril structures, were not included in the fore-mentioned macro-structured 3D models.

Results indicate that the microstructures strongly affect the deformation of skin. Hence, an important challenge involves the modeling of skin microstructure to investigate its effect on mechanoreceptors. Maeno et al. [9] developed a two-dimensional (2D) plane strain model based on cross sections of a human fingertip as opposed to using a whole-fingertip model. Their “intermediate ridges” and fingerprints (also called “papillary ridges”) are based on a 2D model developed by Srinivasan [10]. Maeno et al. found that the SED were more concentrated at the apexes and bases of intermediate ridges. Gerling [11] developed a similar model without fingerprints because fingerprints do not appear to affect the distribution of stress and strain at the tips of intermediate ridges (SA-I locations). The model confirmed that the intermediate ridges might focus SED at the location of SA-I receptors but do not affect the SED distribution.

Meanwhile, scarce attention focused on FA-I receptors, which share the intermediate ridge with SA-I receptors. Current models with or without intermediate ridges tend to focus on positions of the SA-I receptors. The 2D model [9] barely exhibited any effect of the intermediate ridge with respect to the stress concentration at the positions of the FA-I receptors. A potential explanation corresponds to the lack of anatomically detailed dermal papillae in the model.

## The microstructure of skin in three dimensions

A gap exists between the natural geometry and conventional FE models because 2D models assume identical geometry along the depth direction (Fig 1b). In contrast, Cauna’s observation [2] [3] revealed the convex-concave structure of dermal papillae. Additionally, FA-I receptors are located at common apexes of both dermal papillae and the intermediate ridge (Fig 1c), and thus, a 3D model with both intermediate ridges and dermal papillae (3D microstructure) is required to investigate their mechanotransduction.

Vodlak [12] developed a full-scale fingertip with a single representative volume of dermal papillae (containing one FA-I receptor) that focused on the anatomical detail of the FA-I receptor and its transduction method. This model is potentially useful in investigating 3D microstructure although modeling the 3D microstructure at the macro-scale is complex and computationally expensive.

This paper presents a small-scale model that includes the 3D microstructure of dermal papillae. The proposed model is validated with a standard line-load method. This is followed by comparing the 3D model with a model with a 2D microstructure to demonstrate how the 3D microstructure affects the SED concentration at mechanoreceptor positions, i.e., at the base of intermediate ridges (SA-I receptor position) and especially at the tips of dermal papillae (FA-I receptor position). The results indicate that the SED is high at the FA-I receptors near the edge of the indenter in dynamic experiments. During vibrating experiments, the response of SED at a single FA-I location increases when the frequency of vibration increases to 80 Hz.

## Methods

### FE models

**Fig 2:**
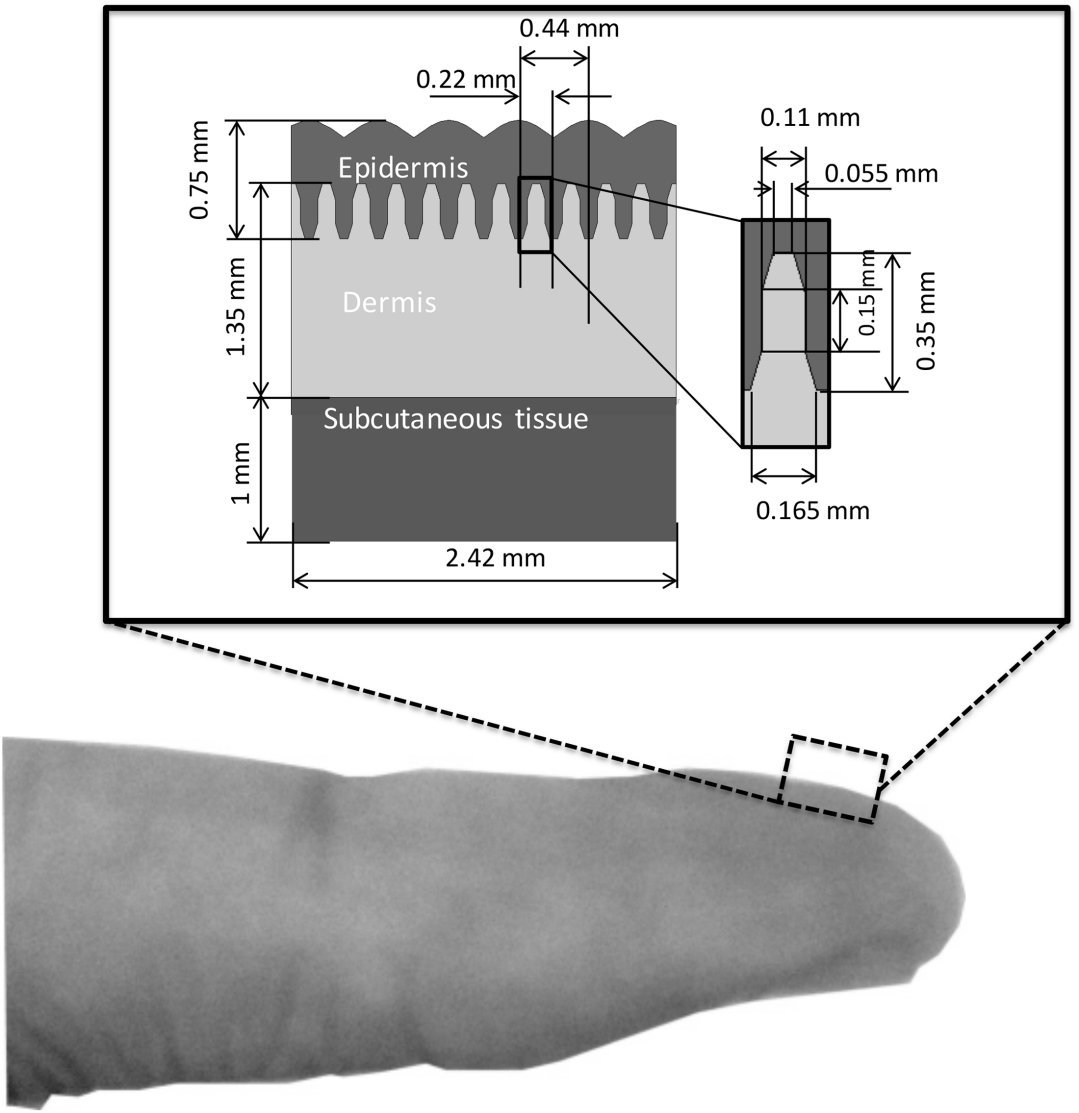
A simplified elastic model mimicking a cut-away cube of the human fingertip. Inset: exterior measurements and measurement of a single intermediate ridge.

The model was considered as a cut-away cube of a fingertip and contained six fingerprints along with 12 intermediate ridges with exterior measurements as shown in Fig 2. The configuration of the limiting ridges is excluded. The model is relatively small, and the fingerprints are arranged evenly. The depth of skin calculated from the surface of the epidermis to the center of bone typically approximately corresponds to 11.55 mm while the convex of the surface is less than 0.121 mm. The difference ratio approximately corresponds to 1.04%, and thus it is reasonable to assume that the surface is flat in this case. This assumption is also supported by anatomical observations [13]. A thin layer of subcutaneous tissue is used as a buffer, and its thickness is determined through a line-load validation as described in the next section. This modeling method reduces the calculation cost while allowing the model to have a sufficient thickness for deformation in three dimensions.

**Fig 3:**
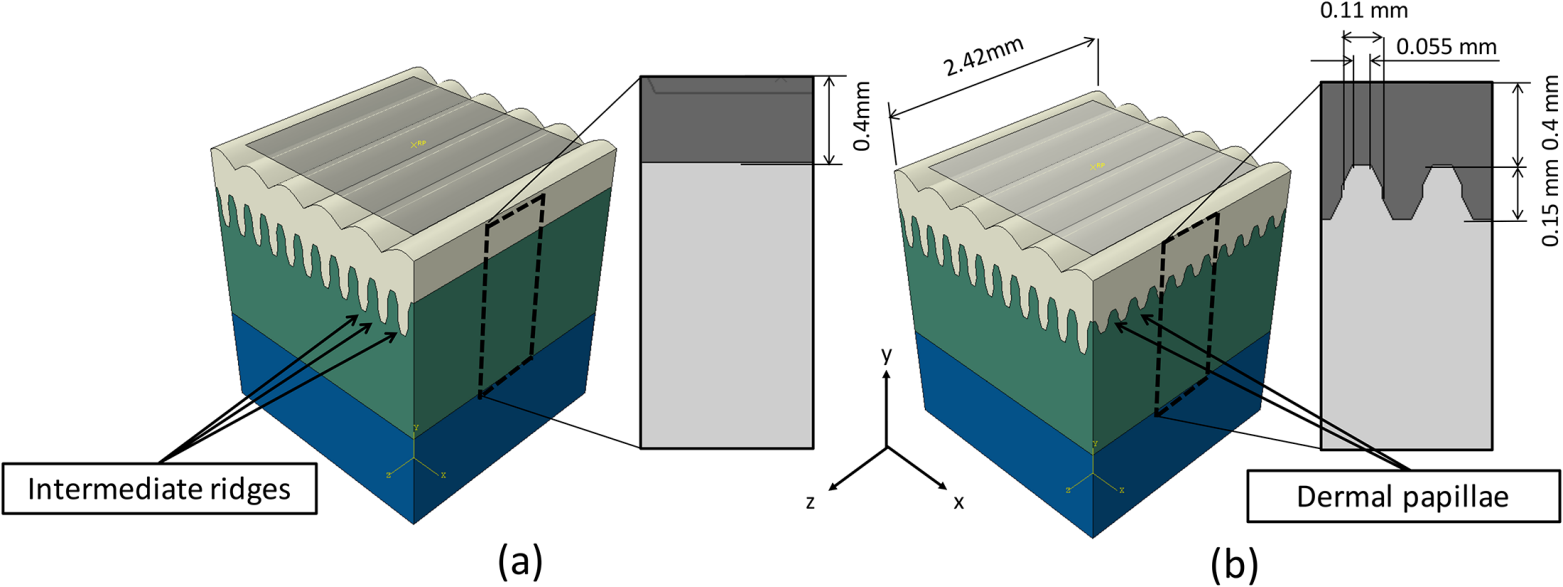
Depth configuration of (a) the 2D ridged model and (b) the 3D ridged model. The epidermis, dermis, and subcutaneous layers are shown in white, green, and blue colors, respectively. The gray plane represents an example of a solid indenter (2×2*mm*^2^). The apexes of dermal papillae exhibit the same structure and measurements as those of intermediate ridges.

Two models were created for the simulation, namely 2D and 3D ridged models. The 2D ridged model includes intermediate ridges, which are concave-convex structures with trapezoid apexes. The dermal papillae are considered to possess even geometry (Fig 3a). The 3D ridged model includes the same intermediate ridges and the concave-convex dermal papillae along the depth (144 tips in total). The dermal papillae are simplified into trapezoid apexes and bases with the same width as those of the intermediate ridge. The length of dermal papillae is set at 0.15 mm, and this is equivalent to the average length of an FA-I receptor in humans (Fig 3b).

The models are multi-layered and include the epidermis, dermis, and subcutaneous tissue (Fig 2) with elastic properties as adapted from previous studies [9]. The Young’s modulus corresponds to 0.136 MPa for the epidermis, 0.08 MPa for the dermis, and 0.034 MPa for the subcutaneous tissue. The Poisson’s ratio corresponds to 0.48 for each layer [9] [11] [14]. In this study, we focus on the mechanical response at FA-I location under small displacement loads, and thus the model is assumed as linearly viscoelastic. Sophisticated hyperelastic models [15] are excluded as [16] [17] suggested that a non-linearity model is necessary for large displacement loads. As suggested by [17], the outer most layer is linear elastic and the others are viscoelastic. The total stress is as follows:

$$\begin{array}{c} \sigma \left(t\right)=\sigma_{0}\left(t\right)+\;\int_{0}^{t}\;\dot{g(\tau )}\sigma_{0}\left(t-\tau \right)d\tau \end{array}$$ (1)

where t denotes time, g(t) denotes the stress relaxation function, and *σ*_0_(*t*) denotes the instantaneous stress. We define g(t) as a two-term Prony series.

$$\begin{array}{c} g\left(t\right)=1-\;\sum_{i=1}^{2}\;g_{i}\left(1-e^{-t/\tau_{i}}\right) \end{array}$$ (2)

where *g*_*i*_ and *τ*_*i*_ denote the stress relaxation parameters that are obtained by fitting the net force response of the model to the experimental data as adopted from [17]. The fitting program is developed and implemented in Python with the support of the Scipy (v0.19.0) library.

The finite element software ANSYS release 16.0 (ANSYS Co.) is used to mesh and analyze the responses. The mesh uses 20-node solid brick elements for surface-to-surface contact. The coefficient of friction between the models and indenters is assumed as zero due to small indentations. The numbers of nodes and elements in 3D ridged models correspond to 267,156 and 88,482, respectively. The numbers of nodes and elements in 2D ridged models correspond to 114,879 and 34,131, respectively. The differences in numbers are due to the complexity of dermal papillae structures in the 3D ridged model. The experiments are conducted in dynamic conditions. Restraint conditions are assumed based on validation experiments that employ various restrained circumstances.

### Viscous parameters calibration for dynamic experiments

**Fig 4:**
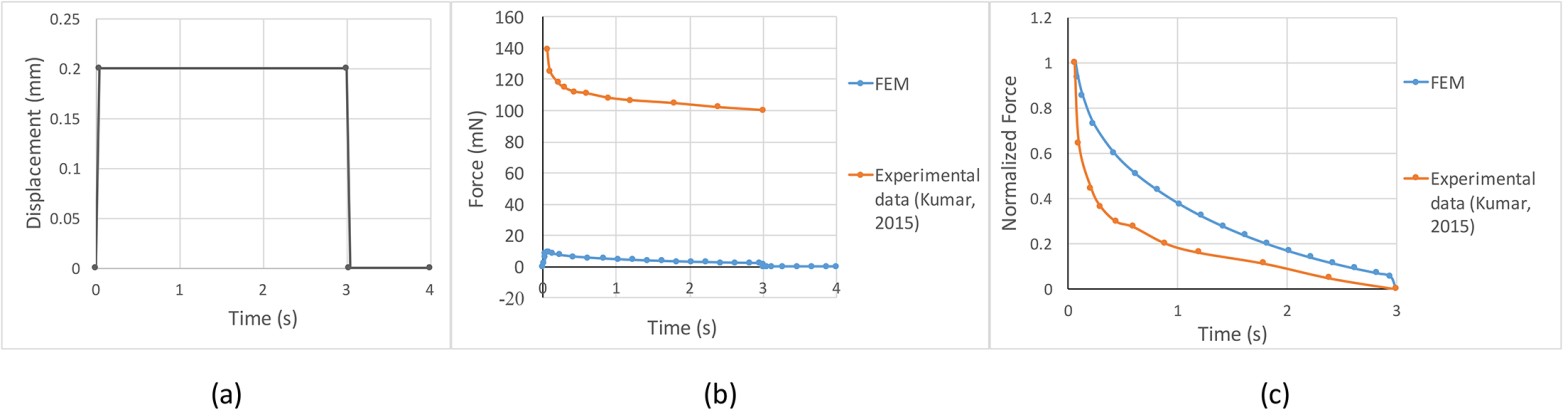
Viscous parameters calibration. (a) The displacement history of an indenter. (b) Profiles of the absolute contact force from the model and experimental data from [17]. (c) Profiles of normalized contact force in relaxation periods showing a good match between the model results and experimental data.

We simulate the indentation experiments with a cylindrical indenter as performed in [17]. The indenter is circular with a diameter of 0.5 mm. The indenter is ramped up to 0.2 mm (0.5 mm/s) and held for 3 s and then completely retracted. We calculate the contact force at the surface of the fingerprints as a function of time. Fig 4a describes the displacement history of indenter, and Fig 4b shows the predicted contact force when compared with the experimental data. A difference in magnitude is expected due to the lack of rigid fixed parts such as bones and nails. Fig 4c shows that the decay of normalized force in our model is similar to that observed by [17].

### Experiments

#### Model validation

**Fig 5:**
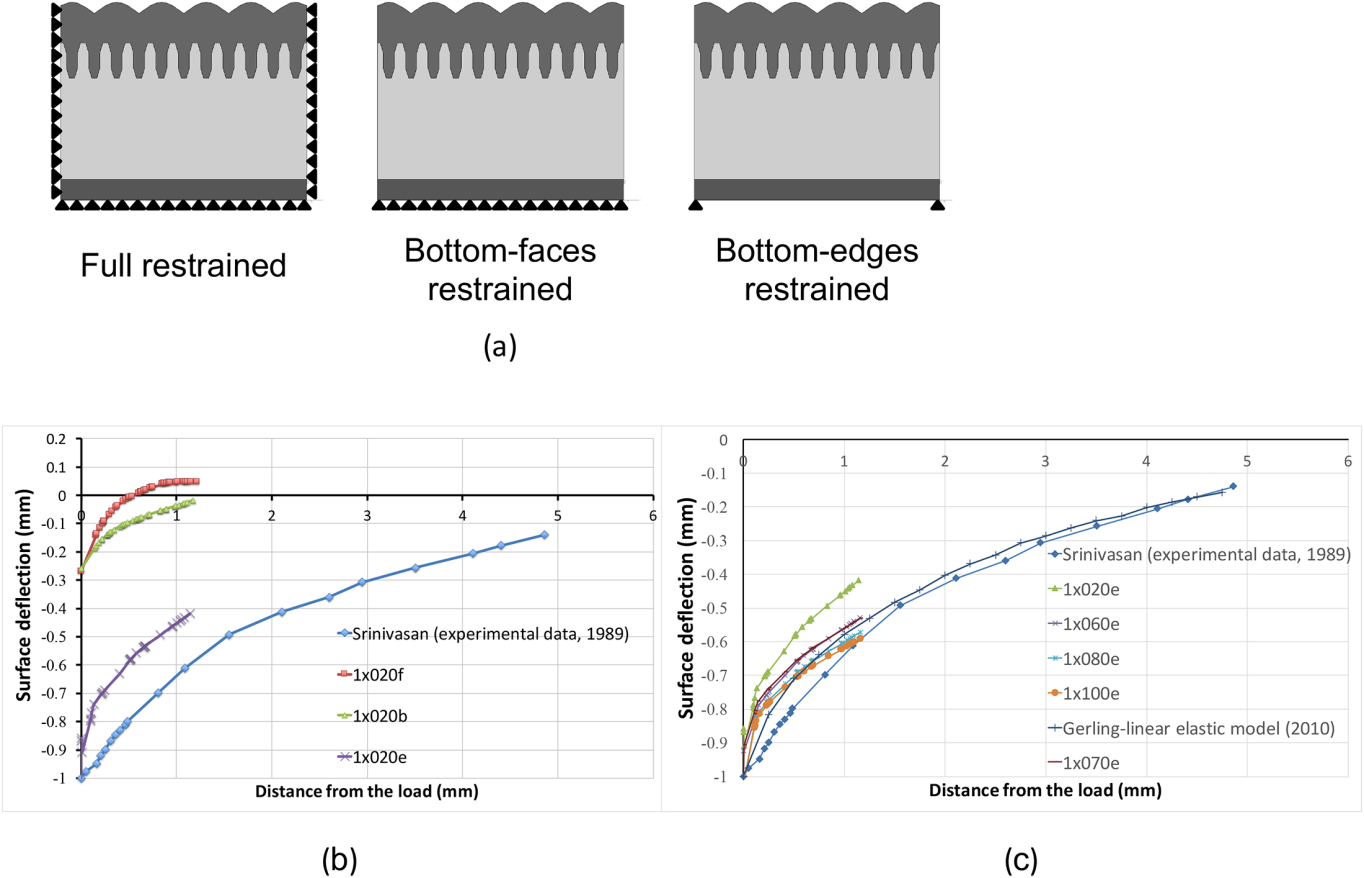
Line load validation test, 50 micrometers. (a) Illustration of three tested restraint conditions. Black triangles represent fixed boundary conditions (an assumption). (b) Surface deflection of the models under each restraint condition when compared with experimental data from Srinivasan et al. (1989). (c) Surface deflection of the models with different thicknesses of cutaneous tissue.

The displacement at the surface of model (in response to 50 *μ*m line load indenter) is compared to experimental data from [7] to validate the mechanical response of the model. The line-load indenter results in a displacement of 1 mm that is perpendicular from the surface of the models (at the rate of 1 mm/s) and is held for 2 s. The surface deflection is computed at t = 2 s to allow the model to reach its stable condition. Three restraint conditions are tested to determine the boundary condition that best approximates the observed displacement behavior, namely full-restrained (bottom and surrounds), bottom-faces-restrained, and bottom-edges-restrained conditions (Fig 5a). The models used for the three boundary conditions are denoted as 1x020s, 1x020f, and 1x020e.

Another boundary test focuses on the thickness of the subcutaneous layer. The thickness of the subcutaneous layer varies in range of 0.2 mm, 0.4 mm, 0.6 mm, 0.7 mm, 0.8 mm, and 1 mm and the six conditions are as denoted 1x020e, 1x040e, 1x060e, 1x070e, 1x080e, and 1x100e, respectively). All the models were bottom-edges-restrained.

#### Ramp-and-hold experiments

In order to investigate the effect of a 3D microstructure, a 2.0×2.0 *mm*^2^ bar provides displacement to a depth of 0.5 mm from the model surface at the rate of 5 mm/s and is held for 2 s. The displayed time point is the first time that the indenter reaches its deepest position (t = 0.1 s). Two 3D distributions of stress are plotted to address the effect in the population. The SED at the position of FA-I receptor was measured at the tips of intermediate ridges (in the 2D ridged model) and the common tips of intermediate ridges and dermal papillae (in the 3D ridged model). The strain rate is constant during the ramp phase, and thus the stress-strain curves are non-linear. For the purpose of convenience, the concept of equivalent strain energy density is used [18]. The convex-concave shape of dermal papillae is considered, and the stress-strain behavior at their tips is similar to the elastic-plastic behavior at a notch tip. The Glinka’s concept relates equivalent strain energy density for an elastic fictitious material as well the real elastic-plastic material during a low strain. Hence, the SED is estimated as follows

$$\begin{array}{c} U=\int \;\sigma d\epsilon \approx \frac{1}{2}\sigma^{e}\epsilon^{e} \end{array}$$ (3)

where *σ*^*e*^ denotes equivalent stress and *ϵ*^*e*^ denotes equivalent strain. The equivalent stress and equivalent strain is related to the principal stresses and strains as given by the following equations

$$\begin{array}{c} \sigma^{e}={\left[\frac{{\left(\sigma_{1}-\sigma_{2}\right)}^{2}+{\left(\sigma_{2}-\sigma_{3}\right)}^{2}+{\left(\sigma_{3}-\sigma_{1}\right)}^{2}}{2}\right]}^{\frac{1}{2}} \end{array}$$ (4)

$$\begin{array}{c} \epsilon^{e}=\frac{1}{1+\nu }{\left(\frac{1}{2}\left[{\left(\epsilon_{1}-\epsilon_{2}\right)}^{2}+{\left(\epsilon_{2}-\epsilon_{3}\right)}^{2}+{\left(\epsilon_{3}-\epsilon_{1}\right)}^{2}\right]\right)}^{\frac{1}{2}} \end{array}$$ (5)

where *ν* denotes the material Poisson’s ratio.

The SED distribution at 10 specific x-axis-crossed surfaces, namely eight in-range and two out-of-range from the indenter for comparison, are recorded and then superimposed.

In order to examine whether the 3D microstructure can help in discerning a similar indenter, we additionally analyze the normalized SED distribution of the following three indenters: a gap indenter (comprising two 0.5×2 *mm*^2^ indenters with a 1.0×2.0 *mm*^2^ gap between inside edges), a 90-degree-rotated gap indenter, and a solid circle indenter (with a diameter of 2 mm). The normalized SED (*ε*_*i*_) at the *i*^*th*^ sample of data is as follows

$$\begin{array}{c} \varepsilon_{i}=\frac{A_{i}-min\left(A\right)}{max(A)-min(A)} \end{array}$$ (6)

where *A* represents the dataset of SED, and *A*_*i*_ denotes the absolute value of SED at the *i*^*th*^ sample. The normalized SED is contoured into a 10 × 10 table with each cell relating to a specific tip of dermal papillae (i.e., 100 tips in total). Only “hot-spots” that correspond to the positions that fall into the right side of the scale bar are plotted; i.e., the threshold corresponds to 0.6. The size is adjusted for better visualization of the distribution as opposed to considering the absolute value.

#### Vibration experiments

The FA-I is sensitive to stimulus in range of 20 to 50 Hz, and thus indentation experiments with four vibrating stimuli (with different frequencies corresponding to 20 Hz, 30 Hz, 50 Hz, and 80 Hz) are conducted. The indenter is a 2×2 *mm*^2^ plane. The amplitude is maintained at 0.5 mm. Previous studies [19] indicated the highest spike rates of FA-I when indenters reached their deepest position. Thus, the frequency-dependent SED response is plotted at a single FA-I near the edge of the indenter at the following time point(e.g., t = 0.005 s for 50 Hz stimuli).

## Results

### Model validation results

Fig 5 shows the predicted surface deflection of skin fitted relative to the experimental data for a human finger. Only half of the profiles are shown due to reasons of symmetry. The maximum displacement of the line-load in the cases of the full-restrained and bottom-face-restrained models is as low as 0.25 mm. The model with the bottom-edge-restrained condition better fits the experimental data as shown in Fig 5b.

The thickness of the subcutaneous layer appears to strongly affect the result of line-load validation. The models with a thickness of the subcutaneous layer ranging from 0.6 mm to 1 mm well fit both the experimental data [7] and the linear-elastic model from [11] as shown in Fig 5c. Each set of 2D and 3D ridged models were treated equally, and thus, the elastic characteristics are similar in both conditions. The 1x100e model (that includes original dimensions with a 1-mm-thick subcutaneous layer; bottom-edge restrained) fit experimental data [7] the most. Hence, the 1x100e model was used in ramp-and-hold experiments and vibration experiments.

### Ramp-and-hold experiment results

**Fig 6:**
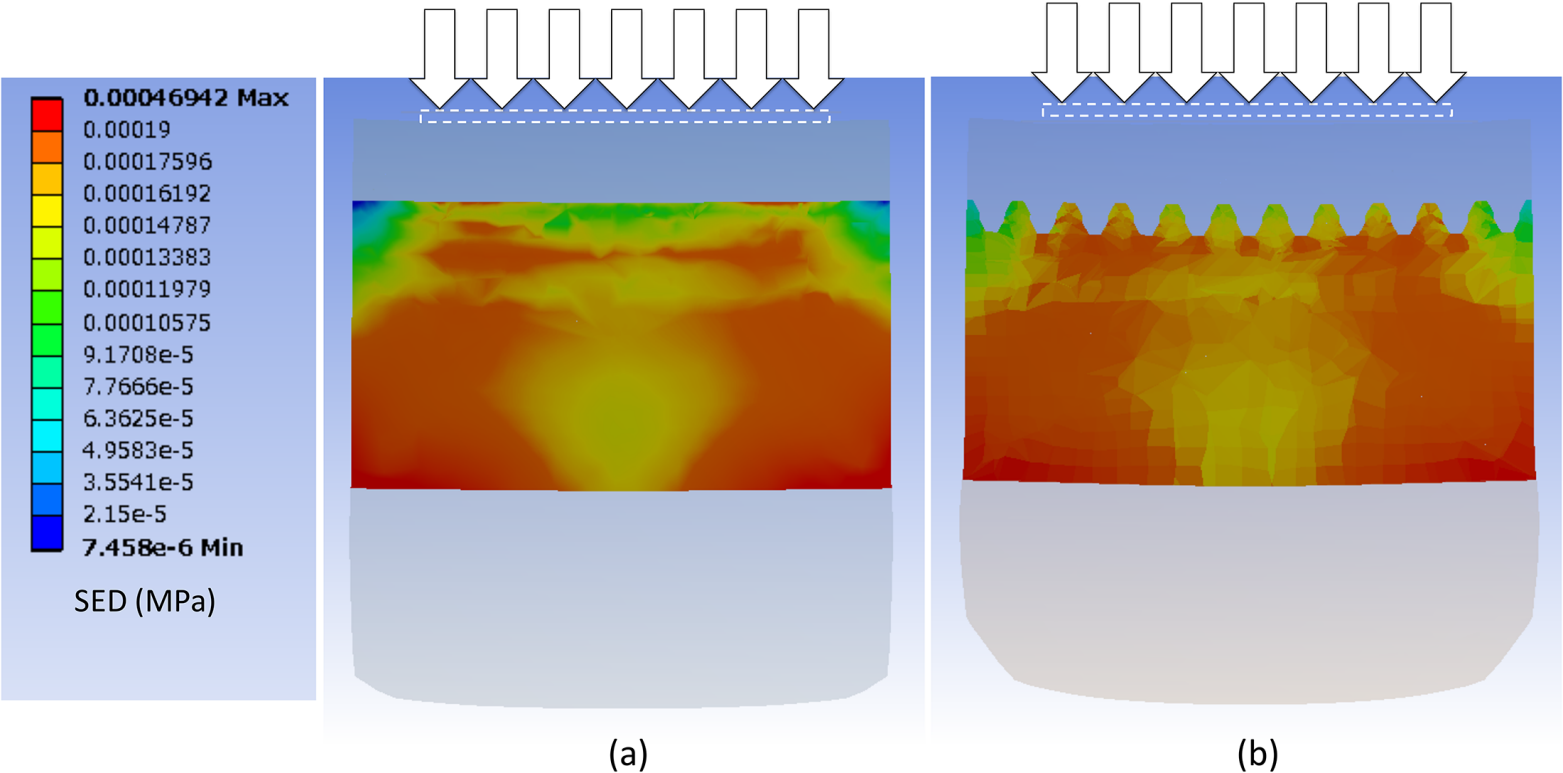
SED distributions along the z-axis of an intermediate ridge. (a) the 2D ridged model and (b) the 3D ridged model. The white square denotes the range of the indenter. Only the dermis is shown. The maximum SED of the scale bar at the left is set at 0.00019 MPa.

Fig 6 illustrates the SED distribution along the z-axis of an intermediate ridge in the case of the bar indenter. The centers of both models only contained the bases of the intermediate ridges, and thus, the observed surface is shifted along the x-axis to the cross section where the SED is highest. The SED is high at the corners of indenters in the 2D ridged model (Fig 6a) although it diffuses separately into the dermal papillae in the 3D ridged model (Fig 6b). A high SED is observed at each tip of the dermal papillae.

**Fig 7:**
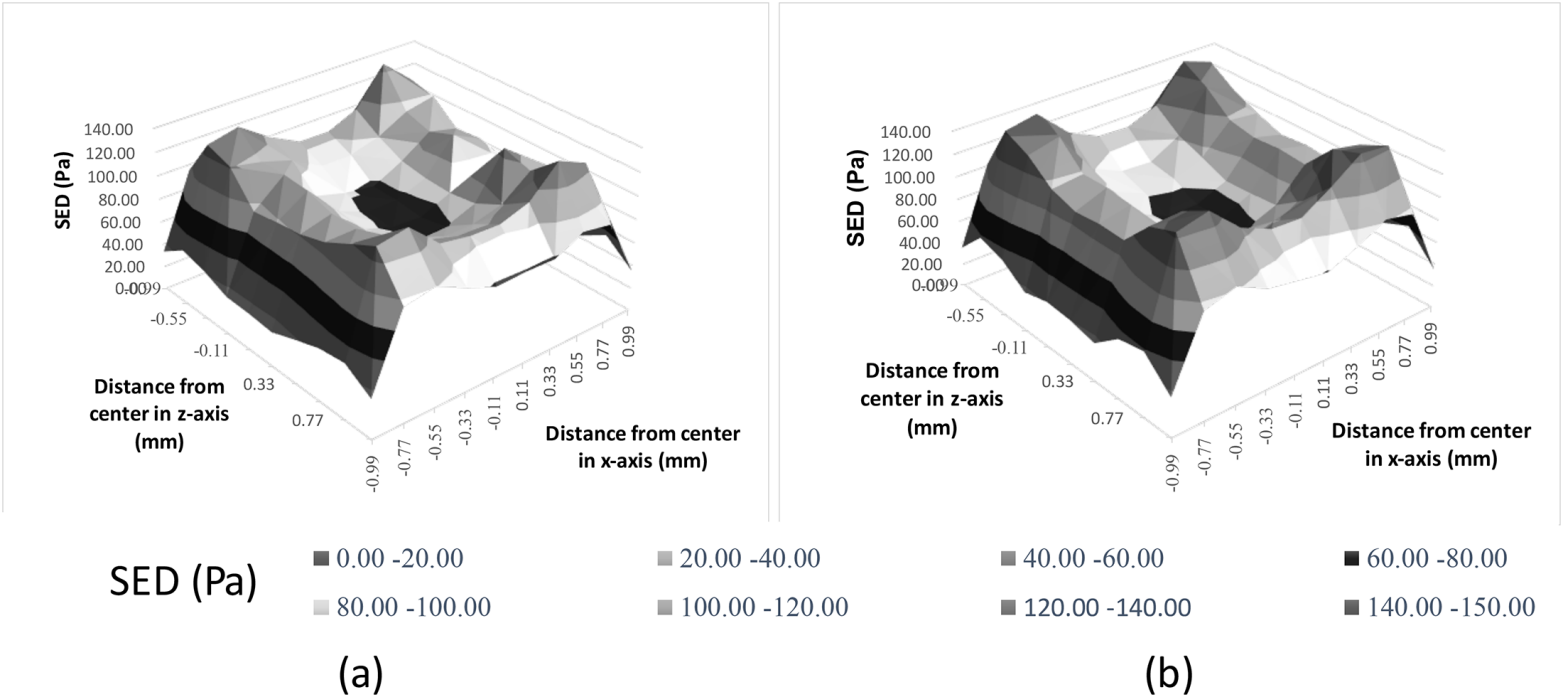
3D distribution of SED at the tips of dermal papillae underlying the indenter. (a) the 2D ridged model and (b) the 3D ridged model.

**Fig 8:**
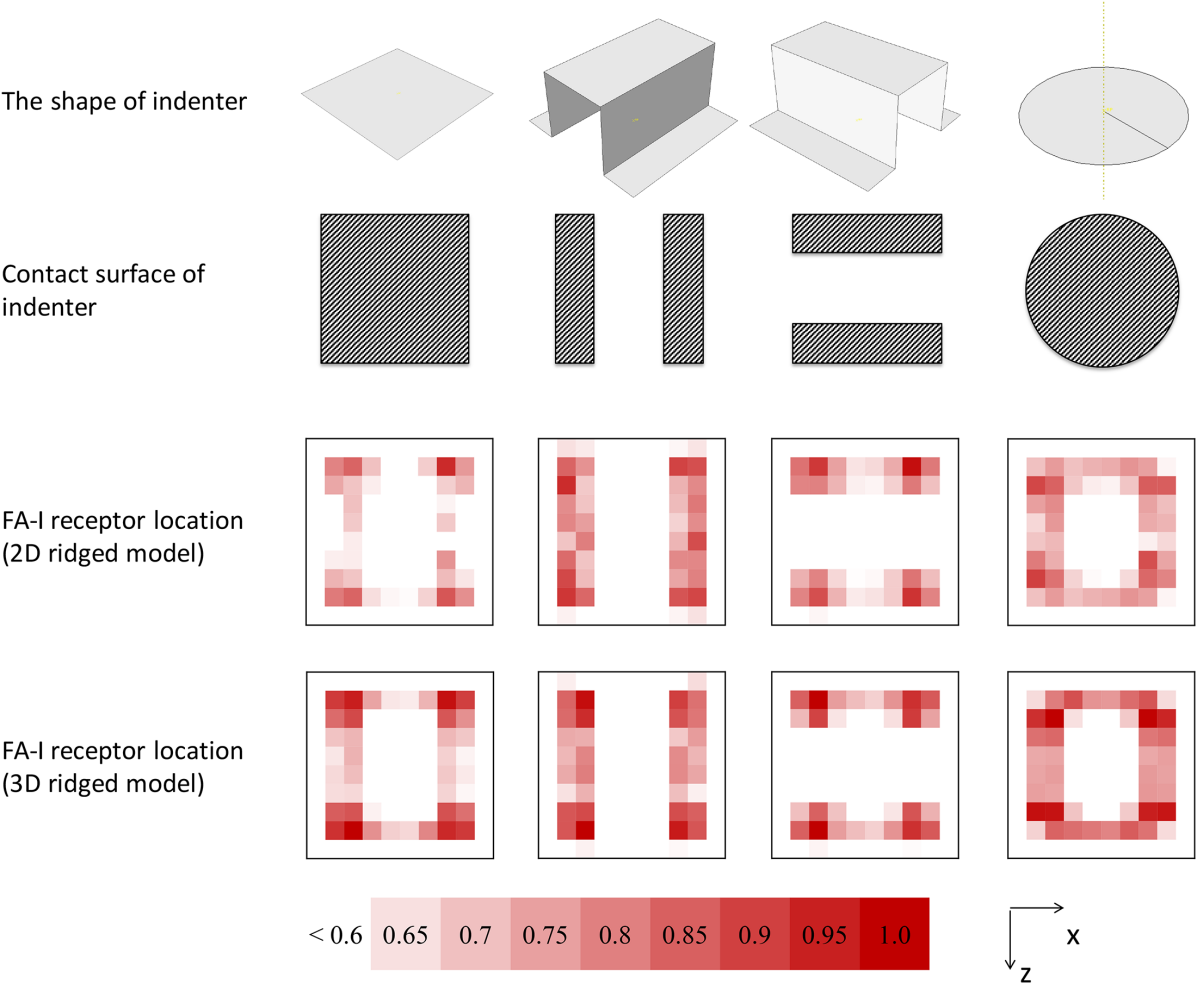
Summary of the normalized SED distribution at the SA-I receptor location and FA-I receptor location, corresponding to the indenter. The indenters are as follows (from left to right): a solid bar indenter, a gap indenter, a 90-degree-rotated gap indenter, and a solid circle indenter. The scale bar shows a gradient from white to red as the normalized SED increases from 0.6 to 1.0. Values under 0.6 are not plotted.

The 3D distribution of SED at the tips of dermal papillae after pressing by the solid bar indenter is shown in Fig 7. The vertical axis presents the magnitude of SED, the horizontal axis presents the distance along the z-axis from the center, and the depth axis presents the distance along the x-axis from the center. The peak value of SED in the 3D ridged model exceeds that in 2D ridged model. A valley is observed at the center of the apex and indicates an interchange zone (peak-valley-peak) in both models. The contour of the normalized SED in the case of solid bar indenter reveals that the interchange zone of the 3D ridged model along the z-direction is more significant than that of the 2D ridged model (Fig 8). Along the x-direction, the interchange zone is equally noticeable in the two models.

A summary of the normalized dataset is shown in Fig 8. In the 2D ridged model, the SED is mostly high at the four corners of the indenters (for example, solid bar indenter and 90-degree-rotated gap indenter) while it mostly high at the edges of the indenters (and especially along the z-axis) in the 3D ridged model. The 3D ridged model produces distributions of the normalized SED at the FA-I receptor location that differentiate the indenters. Furthermore, the 2D ridged model shows little difference between the normalized SED distributions of the solid bar indenter and gap indenter. It is evident that the 3D microstructure significantly influences encoding spatial differences among the indenters.

### Vibration experiment results

**Fig 9:**
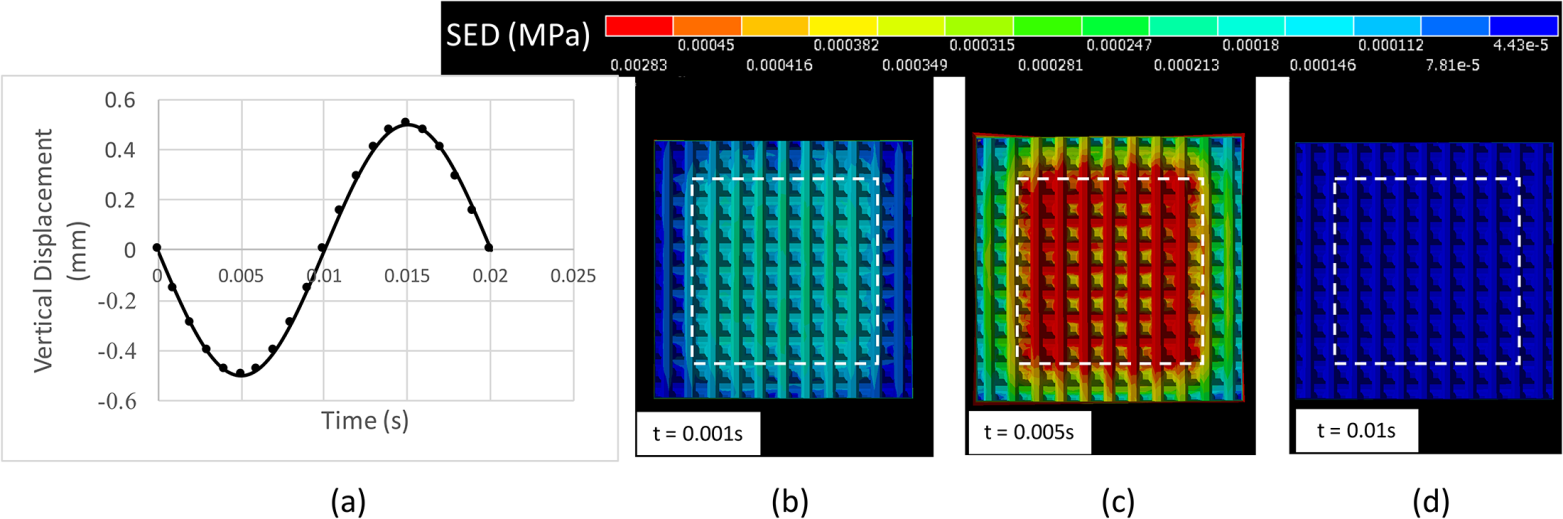
Top view of the dermis part of the 3D ridged model during vibration by a 50-Hz stimuli. (a) Displacement profile of a 50 Hz vibratory indenter. Distribution of SED at FA-I locations at (b) t = 0.001 s, (c) t = 0.005 s, and (d)t = 0.1 s. The white square depicts the range of the indenter.

Fig 9b, 9c and 9d show the SED distribution at a population of FA-I location during vibration by 50 Hz stimuli. The displayed time-points correspond to 0.001 s, 0.005 s, and 0.01 s. The observed model is a 3D ridged model. The localizing effect in which the SED is high near the edges of the indenter is observed at t = 0.005 s. The localizing effect is unlikely to be observed at the other time-points.

**Fig 10:**
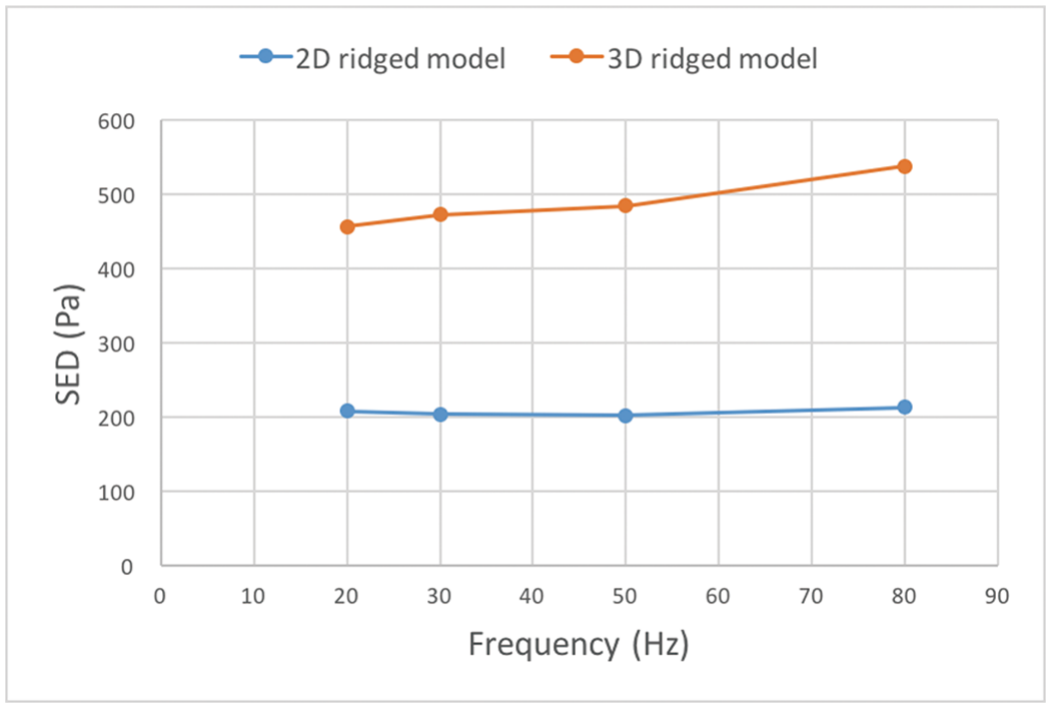
SED response at a FA-I location as a function of frequency, and a comparison of the 3D model and the 2D model.

In order to examine the change of SED when the frequency increases, the SED at a FA-I location (with the maximum SED value) is plotted and compared with the following position in the 2D ridged model (Fig 10). In the 3D ridged model, the SED increases when the frequency increases to 80 Hz while it remains constant in the 2D ridged model.

## Discussion

Previous studies showed the effect of microstructure on stress/strain behavior at the position of mechanoreceptors (SA-I and FA-I) where the microstructure amplifies the SED concentration at the SA-I receptor’s location. However, most studies were conducted in two dimensions since they lack the depth configuration of dermal papillae and overlooked the effect of the FA-I receptor positions. The present study involved proposing and developing a model that presents the 3D microstructure of skin in significant detail (i.e., a 3D ridged model). The boundary conditions were determined through a validation experiment with a standard line load method (Fig 5). The viscous parameters were determined by fitting the normalized contact force on the circular indenter to the experimental data reported by [17]. The approach reduces the calculation cost while ensuring that the performance of the model is comparable to that of other models. The limitations of the model will be discussed in the final part of this section.

Our main finding indicates that the 3D microstructure appears to modify the distribution of SED at FA-I receptor’s positions such that it helps in discerning the spatial configuration of similar indenters (Fig 8). This finding agrees and interprets the conclusion of a recent study by Harih et al. [20] in which the 2D and 3D fingertip FE models were justified during static contact simulation. Although the results of the 2D and 3D model in [20] were similar (possibly due to the lack of microstructure), the 3D model provides additional insights into the third dimension. The results suggest that the 3D microstructure provides focalizing effects and especially localizing effects as described below.

### The diffusion of SED and the focalizing effect

The focalizing effect describes the trend of SED concentration at the tips of dermal papillae (FA-I receptor’s positions). The effect occurs when the contact surface exhibits uneven geometry and the stiffness of the epidermis and dermis are significantly different. Gerling [21] demonstrated the lensing effect of ridged geometry in a 2D model of a microstructure and concluded that stress is more concentrated at the bases of intermediate ridges. In the 3D model of the microstructure, we confirmed a similar effect given that a high concentration of SED was present. at the tips of the dermal papillae in Fig 6.

### Localizing effect of 3D microstructure and its potential roles in spatial discrimination

The localizing effect describes the peaks of the 3D distribution of SED located at the tips of dermal papillae near the edges of the indenter (Fig 7). The contrast between these positions and others produced a specific pattern that reflects the shape and the size of the indenter (Fig 8). The localizing effect was confirmed in both ramp-and-hold experiments (Fig 8 and S1 Fig) and vibration experiments (Fig 9) at the moment when the indenters reached their deepest positions.

The 3D ridged model appears to be sensitive to the edges of the indenter with a valley of SED values at the center. In the case of the solid bar indenter, the interchange zone in the x-axis (peak–valley–peak) is more noticeable than that in the z-axis (Fig 8). This behavior may be related to the presence of fingerprints along the x-axis given that the fingerprints are believed to affect the stress/strain concentration at the dermal papillae as shown in [9].

Interestingly, the normalized SED distributions at the FA-I receptor location were sensitive to the edges of indenters in most experiments (see Fig 8). This finding does not contradict the hypothesis that the SA-I receptors are more sensitive than FA-I receptors in terms of shape discrimination (for details, see [5, 22]). However, it indicates that the mechanical response at FA-I receptor location might provide complementary information for better discrimination under dynamic conditions.

### Effect of 3D microstructure in vibratory conditions

In the 3D model of the microstructure, the SED response at a FA-I location increased when the vibratory frequency increased from 20 Hz to 80 Hz (Fig 10), irrespective of the widely-known maximum sensitive frequency of the FA-I receptor (50 Hz). The increase is understandable since the viscous force is proportional to velocity. In this study, the amplitude was kept constant in all experiments, and thus the higher frequency stimulus is displaced at a higher velocity.

Interestingly, the SED response at a FA-I location in the 2D ridged model remained unchanged (Fig 10). Evidently, either the increase was extremely small in the 2D ridged model or the energy was further concentrated at other positions, e.g. SA-I locations. Meanwhile, in the 3D ridged model, the increase in SED response at a FA-I location was high due to the focalizing effect. This result indicates the requirement for a 3D model of microstructure for FA-I related analysis in vibratory conditions.

**Fig 11:**
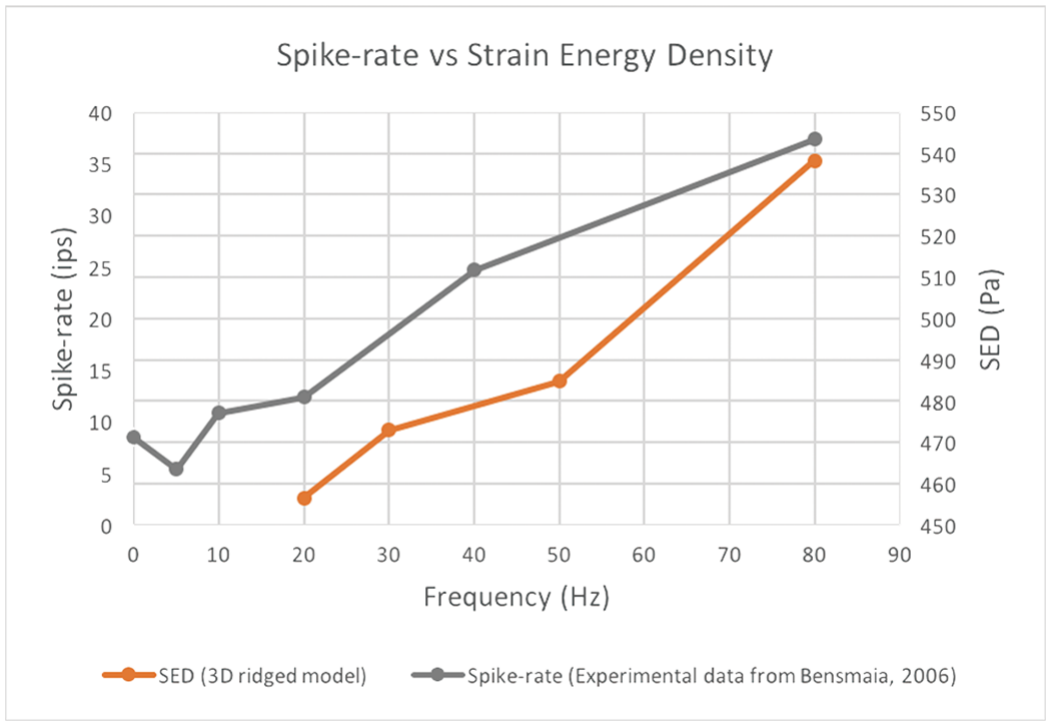
Comparison of 3D model and the mean spike rates of FA-I, adapted from [23].

An electrophysiological report published by Bensmaia [23] showed that the spike rates also gradually increased from 20 Hz to 80 Hz, and this was similar to the SED behavior observed in our study (Fig 11). This result is in agreement with the results obtained by previous studies that suggested a relationship between the dynamic spike rate and local SED at a mechanoreceptor’s location [17] (in this case, this is at the FA-I location).

### The potential use of 3D microstructure in tactile sensor design

In terms of tactile sensor design, the elastic cover is highly essential to protect the sensor from being damaged by external forces [24]. However, Shimojo [25] demonstrated that the elastic cover significantly reduced the spatial resolution of the sensor even if the cover thickness is only 0.2 mm. Various studies mimicked the natural geometry of human finger microstructure in sensor design to improve tactile sensing ability while retaining protection such as studies that indicated that artificial epidermal ridges enhanced strain gage sensitivity by 1.8 times when compared with tactile sensors without ridges [26] or enhanced tactile shape discrimination [27, 28]. An optical tactile sensor developed by Chorley et al. [29] with an array of identical urethane pins as artificial dermal papillae were capable of encoding edge information.

Here, we propose the potential use of a 3D microstructure in tactile sensor design. Given the assumption that the sensing elements respond equally, placing the same at different positions of 3D microstructure allows us to achieve different effects intentionally. The positions at the tips of dermal papillae enhance the spatial discrimination of the tactile sensor (our finding) while the positions at the bases of the intermediate ridges enhance the sensitivity (as suggested in [11]). The contour of the 3D ridged model in Fig 8 implies the tactile image obtained by embedding sensing elements at the tips of dermal papillae under the following circumstances.

### The limitations of proposed model and future works

The limitation of the model is that the model is small, and this restricts the size of the indenters. The current model cannot be used to show the response at sides (left-right) of the fingertip. It is necessary to develop a large-scale model with respect to the complexity and computational cost to facilitate comparisons with physiological or electrophysiological experiments. Physiological or electrophysiological experiments are usually conducted with indenters larger than 2 mm. In this study, the neuronal responses of FA-I mechanoreceptors were not examined due to the lack of a neuronal model. Additionally, the neuronal response of FA-I mechanoreceptor is suggested as a combination of population activities [23, 30]. It is unknown as to precisely how the FA-I mechanoreceptor modifies responses during the combination. Hence, the determination of the manner in which the FA-I mechanoreceptor processes the population mechanical responses into neural responses is an interesting challenge for future work. To investigate the neuronal responses of FA-I mechanoreceptors, one could use a transduction function to transform the SED into neural current. The transduction function was assumed as a sigmoidal stimulus-current curves in [31], and as a two linear function in [6]. Then the spike times could be obtained by using the leaky integrate-and-fire model.

## Conclusion

We developed a model with the 3D microstructure of skin and compared it with conventional 2D microstructure to examine the effect of natural configuration on the SED distribution of mechanoreceptors. The analysis was conducted with various types of indenters under dynamic conditions. The results indicate that the 3D microstructure modifies the SED at FA-I mechanoreceptors such that it helps in discerning similar indenters. The 3D microstructure enhanced the SED response at a FA-I mechanoreceptor when the stimulus frequency increased.

The cover design of available tactile sensors would benefit from our findings. The model produced a specific response pattern of SEDs corresponding to each indenter. This feature along with the currently popular neural sub-model may allow us to effectively discriminate between similar indenters.

## Supporting information

S1 Fig Top view of dermis part of the 3D ridged model during ramp-and-hold experiment. (a) Displacement profile of solid bar indenter. Distribution of SED at FA-I locations at (b) t = 0.01 s, (c) t = 0.1 s, and (d)t = 2 s. The white square is the range of indenter.
(TIF)

## References

[1] Wigley C. Skin and its appendages In: Standring S, Borley NR, editor. Gray’s Anatomy: The anatomical basis of clinical practice. 14th ed London: Elsevier; 2008 p. 145–157.

[2] Cauna N. Nature and functions of the papillary ridges of the digital skin. Anat Rec. 1954;119:449–468. doi: 10.1002/ar.1091190405 13283320 10.1002/ar.1091190405

[3] Cauna N, Mannan G. Organization and development of the preterminal nerve pattern in the palmar digital tissues of man. J Comp Neur. 1961;117:309–328. doi: 10.1002/cne.901170304 13877427 10.1002/cne.901170304

[4] Dandekar K, Raji BI, Srinivasan MA. 3-D Finite element models of human and monkey fingertips to investigate the mechanics of tactile sense. ASME J Biomech Eng. 2003;125(5):682–691. doi: 10.1115/1.1613673 10.1115/1.1613673 14618927

[5] Phillips JR, Johnson KO. Tactile spatial resolution. II. Neural. Representation of bars, edges, and gratings in monkey primary afferents. J Neurophysiol. 1981;46(6):1192–1203. 6275041 10.1152/jn.1981.46.6.1192

[6] Gerling GJ, Rivest II, Lesniak DR, Scanlon JR, Wan L. Validating a population model of tactile mechanotransduction of slowly adapting type I afferents at levels of skin mechanics, single-unit Response and psychophysics. IEEE Trans Haptics. 2014;7(2):216–228. doi: 10.1109/TOH.2013.36 24960553 10.1109/TOH.2013.36 PMC4300237

[7] Srinivasan MA. Surface deflection of primate fingertip under line load. J Biomech. 1989;22(4):343–349. doi: 10.1016/0021-9290(89)90048-1 2745468 10.1016/0021-9290(89)90048-1

[8] Goodwin AW, John KT, Marceglia AH. Tactile discrimination of curvature by humans using only cutaneous information from the fingerpads. Exp Brain Res. 1991;86(3):663–672. doi: 10.1007/BF00230540 1761098 10.1007/BF00230540

[9] Maeno T, Kobayashi K, Yamazaki N. Relationship between the structure of human finger tissue and the location of tactile receptors. JSME Int J. 1997;41:94–100. doi: 10.1299/jsmec.41.94

[10] Srinivasan MA, Dandekar K. An investigation of the mechanics of tactile sense using two-dimensional models of the primate fingertip. J Biomech Eng. 1996;118(1):48–55. doi: 10.1115/1.2795945 8833074 10.1115/1.2795945

[11] Gerling GJ. SA-I mechanoreceptor position in fingertip skin may impact sensitivity to edge stimuli. Appl Bionics Biomech. 2010;7(1):19–29. doi: 10.1155/2010/874936

[12] Vodlak T, Vidrih Z, Pirih P, Skorjanc A, Presern J, Rodic T. Functional microanatomical Model of Meissner corpuscle—From finite element model to mechano-transduction In: Auvray M, Duriez C, editors. EuroHaptics (2). vol. 8619 of Lecture Notes in Computer Science. Springer; 2014 p. 377–384.

[13] Adams MJ, Johnson SA, Lefèvre P, Lévesque V, Hayward V, André T, et al Finger pad friction and its role in grip and touch J R Soc Interface. 2013;10(80). 10.1098/rsif.2012.0467 PMC3565724 23256185

[14] Fung YC. Biomechanics-mechanical properties of living tissue. 2nd ed Springer-Verlag; 1993.

[15] Wu JZ, Dong RG, Rakheja S, Schopper AW, Smutz WP. A structural fingertip model for simulating of the biomechanics of tactile sensation. Med Eng Phys. 2004;26(2):165—175. doi: 10.1016/j.medengphy.2003.09.004 15036184 10.1016/j.medengphy.2003.09.004

[16] Ge W, Khalsa PS. Encoding of compressive stress during indentation by slowly adapting type-I mechanoreceptors in rat hairy skin. J Neurophysiol. 2002;87(4):1686–1693. doi: 10.1152/jn.00414.2001 11929890 10.1152/jn.00414.2001

[17] Kumar S, Liu G, Schloerb DW, Srinivasan MA. Viscoelastic characterization of the primate finger pad in vivo by microstep indentation and three-dimensional finite element models for tactile sensation Studies. ASME J Biomech Eng. 2015;137(6):061002–061002–10. doi: 10.1115/1.4029985 10.1115/1.4029985 PMC4403516 25751365

[18] Molski K, Glinka G. A method of elastic-plastic stress and strain calculation at a notch root. Mater Sci Eng. 1981;50(1):93–100. doi: 10.1016/0025-5416(81)90089-6

[19] Talbot WH, Smith ID, Kornhuber HH, Mountcastle VB. The sense of flutter-vibration: comparison of the human capacity with response patterns of mechanoreceptive afferents from the monkey hand. J Neurophysiol. 1968;31(2):301–334. 4972033 10.1152/jn.1968.31.2.301

[20] Harih G, Tada M, Dolšak B. Justification for a 2D versus 3D fingertip finite element model during static contact simulations. Comput Methods Biomech Biomed Engin. 2016;19(13):1409–1417. doi: 10.1080/10255842.2016.1146712 26856769 10.1080/10255842.2016.1146712

[21] Gerling GJ, Thomas GW. The effect of fingertip microstructures on tactile edge perception. In: Proc. First Joint Eurohaptics Conf. Symp. Haptic Interfaces Virtual Environ. Teleoperator Syst; 2005. p. 63–72.

[22] Blake DT, Johnson KO, Hsiao SS. Monkey cutaneous SA-I and RA responses to raised and depressed scanned patterns: effects of width, height, orientation, and a raised surround. J Neurophysiol. 1997;78(5):2503–2517. 9356401 10.1152/jn.1997.78.5.2503

[23] Bensmaia SJ, Craig JC, Yoshioka T, Johnson KO. SA1 and RA afferent responses to static and vibrating gratings. J Neurophysiol. 2006;95(3):1771–1782. doi: 10.1152/jn.00877.2005 16236779 10.1152/jn.00877.2005 PMC1839046

[24] Dahiya RS, Metta G, Valle M, Sandini G. Tactile sensing- from humans to humanoids. IEEE Trans Robotics. 2010;26(1):1–20. doi: 10.1109/TRO.2009.2033627

[25] Shimojo M. Spatial filtering characteristic of elastic cover for tactile sensor. In: IEEE Proc. of Robotics and Automation; 1994. p. 287–292.

[26] Zhang Y, Miki N. Sensitivity enhancement of a micro-scale biomimetic tactile sensor with epidermal ridges. J Micromech Microeng. 2010;20(12):129801 doi: 10.1088/0960-1317/20/12/129801

[27] Salehi S, Cabibihan JJ, Ge SS. Artificial skin ridges enhance local tactile shape discrimination. Sensors. 2010;11:8626–8642. doi: 10.3390/s110908626 10.3390/s110908626 PMC3231512 22164095

[28] Vasarhelyi G, Adam M, Vazsonyi E, Barsony I, Ducso C. Effects of the elastic cover on tactile sensor arrays. Sens Actuat A Phys. 2006;132:245–251. doi: 10.1016/j.sna.2006.01.009

[29] Chorley C, Melhuish C, Pipe T, Rossiter J. Development of a tactile sensor based on biologically inspired edge encoding. In: Proc. of IEEE International Conference on Advanced Robotics; 2009. p. 1–6.

[30] Pare M, Smith A, Rice F. Distribution and terminal arborizations of cutaneous mechanoreceptors in the glabrous finger pads of the monkey. J Comp Neur. 2002;445:347–359. doi: 10.1002/cne.10196 11920712 10.1002/cne.10196

[31] Lesniak DR, Gerling GJ. Predicting SA-I mechanoreceptor spike times with a skin-neuron model. Math Biosci. 2009;220(1):15–23. doi: 10.1016/j.mbs.2009.03.007 19362097 10.1016/j.mbs.2009.03.007 PMC2754744

