# Supplementary figures and images for: Effect of 3D microstructure of dermal papillae on SED concentration at a mechanoreceptor location
Source: PLoS One. 2017 Dec 8;12(12):e0189293. doi: 10.1371/journal.pone.0189293 (PMC5722322; doi:10.1371/journal.pone.0189293)

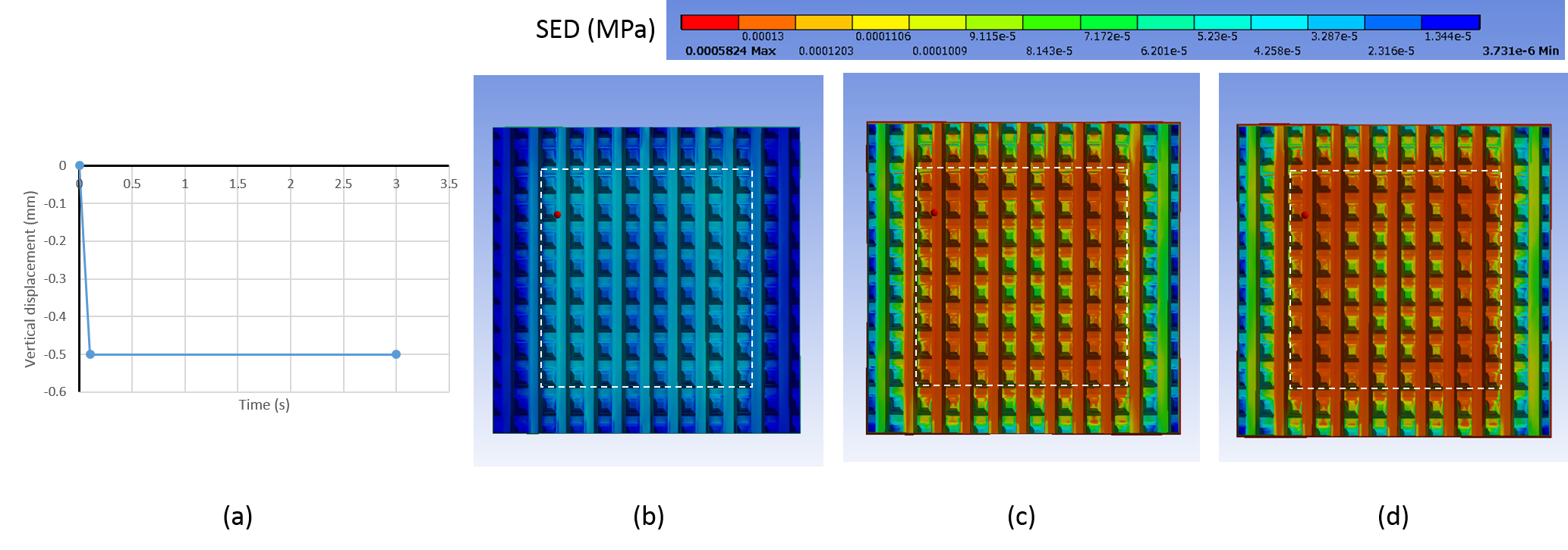

Supplement: S1 Fig — (a) Displacement profile of solid bar indenter. Distribution of SED at FA-I locations at (b) t = 0.01 s, (c) t = 0.1 s, and (d)t = 2 s. The white square is the range of indenter. (TIF) [file pone.0189293.s001.tif]
